# Role of NLRP3 Inflammasome Activation in Obesity-Mediated Metabolic Disorders

**DOI:** 10.3390/ijerph18020511

**Published:** 2021-01-10

**Authors:** Kaiser Wani, Hind AlHarthi, Amani Alghamdi, Shaun Sabico, Nasser M. Al-Daghri

**Affiliations:** Biochemistry Department, College of Science, King Saud University, Riyadh 11451, Saudi Arabia; kwani@ksu.edu.sa (K.W.); hindalharthi1@gmail.com (H.A.); aalghamedi@ksu.edu.sa (A.A.); ssabico@ksu.edu.sa (S.S.)

**Keywords:** NLRP3 inflammasome, metabolic stress, insulin resistance, diabetes, obesity

## Abstract

NLRP3 inflammasome is one of the multimeric protein complexes of the nucleotide-binding domain, leucine-rich repeat (NLR)-containing pyrin and HIN domain family (PYHIN). When activated, NLRP3 inflammasome triggers the release of pro-inflammatory interleukins (IL)-1β and IL-18, an essential step in innate immune response; however, defective checkpoints in inflammasome activation may lead to autoimmune, autoinflammatory, and metabolic disorders. Among the consequences of NLRP3 inflammasome activation is systemic chronic low-grade inflammation, a cardinal feature of obesity and insulin resistance. Understanding the mechanisms involved in the regulation of NLRP3 inflammasome in adipose tissue may help in the development of specific inhibitors for the treatment and prevention of obesity-mediated metabolic diseases. In this narrative review, the current understanding of NLRP3 inflammasome activation and regulation is highlighted, including its putative roles in adipose tissue dysfunction and insulin resistance. Specific inhibitors of NLRP3 inflammasome activation which can potentially be used to treat metabolic disorders are also discussed.

## 1. Introduction

The innate immune system, especially macrophages, plays a central role in the host’s defense against infections and damaged tissues. The activation of downstream signaling cascades and immune responses initiated in immune cells such as macrophage and dendritic cells, triggers infection by the recognition of germline-encoded pattern recognition receptors (PRR), which recognize pathogen- and danger-associated molecular patterns (PAMPs and DAMPs) [1]. PAMPs are derived from microorganisms, such as bacterial endotoxin, and DAMPs are derived from host cells, including tumor cells, dead cells, and products released in response to signals [2]. DAMPs related to metabolic disorders could initiate a pro-inflammatory response by using a wide range of PRRs. They are divided into two main groups: membrane-bound PRRs, such as toll-like receptors (TLRs) and C-type lectin receptors (CLRs), and cytoplasmic PRRs, such as RIG-I-like receptors (RLRs). Inflammasomes are cytoplasmic pattern recognition receptors which are present in several cell types and have been implicated in recognizing endogenous danger signals, leading to the development of inflammation [3]. Inflammasomes, first discovered in 2002 [4], are large multiprotein complexes stimulated by several factors that lead to resolution of infection but can also participate in the pathology of cancer, inflammatory disorders, autoimmune disorders, and infectious diseases [5,6]. Of particular importance is NLRP3 (pyrin domain containing 3) inflammasome from nucleotide-binding oligomerization domain-like receptor (NLR) family which consists of NLRP3, apoptosis-related speck-like protein (ASC), and caspase-1. The other lesser known members of NLR family are NLRP1, NLRP2, NLRP4 to NLRP14 which comprises of 22 NLR’s known till date including NLRP3 [7]. NLRs are classified and named according to their domain structure. NLR proteins have three conserved domains: a central nucleotide-binding and oligomerization domain (NACHT), a C-terminal leucine-rich repeat domain (LRR), and an N-terminal effector domain as N-terminal pyrin (PYD) forming NLRP subgroup. In ASC, PYD associates with a large N-terminal protein-protein interaction motif known as caspase activation recruitment domain (CARD), crucial for the formation of inflammasomes [8]. The assembling of NLRP3 inflammasome leads to the activation of caspase-1-mediated inflammatory responses, including cleavage secretion of the proinflammatory cytokines-IL-1β and IL-18 and the initiation of an inflammatory form of cell death referred to as pyroptosis [9].

Metabolic syndrome (MetS) is a state of steady, systemic low-grade inflammation, and adipose tissue plays an important role in its development [10]. Classically, inflammation occurs as a result of immune cells acting on the invading pathogens. However, due to the development of age-related chronic diseases such as obesity in adulthood, immune cells may alter many pathways, forming an interface termed ”immunometabolism” and resulting in local or systemic inflammation which disrupts homeostasis [11,12]. White adipose tissue (WAT), normally an active endocrine organ, regulates systemic metabolism by secreting various cell-signaling proteins called adipokines [13]. This secretome of WAT, in response to excessive caloric intake in obesity, is altered greatly [14], activates adipose tissue macrophages (ATM) and shifts from anti-inflammatory M2 to pro-inflammatory M1 type [15]. Interaction between these invading ATM’s and metabolic cells (i.e., adipocytes) promotes metabolic stress. Metabolic stress and resulting DAMP’s engage PRRs of the innate immune system, thus triggering pro-inflammatory and stress pathways in the adipose tissue through the activation of cytosolic oligomer complexes called NLRP3 inflammasomes [16].

Obesity-induced inflammation may lead to disorders in lipid and glucose metabolic pathways, such as insulin resistance, diabetes, and atherosclerosis [17,18]. The production of pro-inflammatory cytokines, including interleukin 1β (IL-1β) and interleukin 18 (IL-18), is mediated by the activation of cytosolic multiprotein oligomers of the innate immune system called inflammasomes [19,20,21], classified according to the specific scaffold domains with similar or different biological effects. Among these inflammasomes, the most intensively studied is NLRP3 inflammasome. Its activation is induced by multiple stimuli leading to a cascade of pro-inflammatory processes which if unchecked may lead to systemic inflammation. This obesity-mediated inflammation through NLRP3 inflammasome results in a further deterioration of metabolic control leading to metabolic disorders such as non-alcoholic fatty liver disease (NAFLD) [22]. Conversely, its inactivation by inhibitors significantly alleviates metabolic disorders [23]. This narrative review focuses on NLRP3 inflammasome activation as the mediator of systemic inflammation in obesity and metabolic disorder and factors that regulate this activation in adipose tissue. Understanding the promoters and inhibitors involved in this inflammation activation pathway may help to devise preventive strategies for obesity-mediated inflammation-driven metabolic diseases. 

## 2. NLRP3 Inflammasome Activation in Adipose Tissue

**Figure 1:**
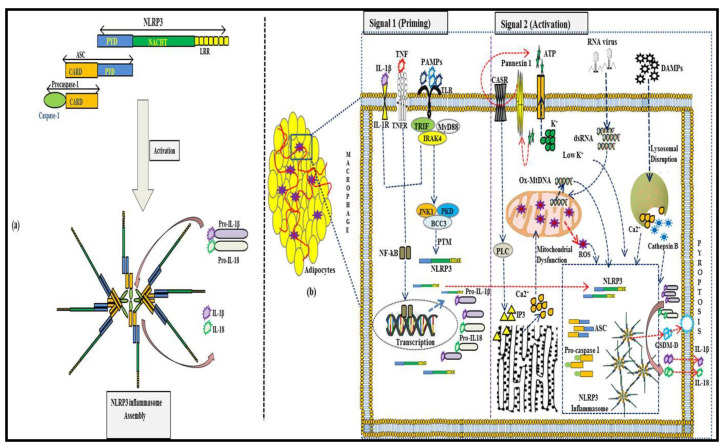
Classical NLRP3-inflammasome activation mediated maturation of pro-inflammatory signals in adipose tissue. (**a**): Individual components of NLRP3 inflammasome. It consists of NLRP3 protein with leucine-rich repeats (LRR), globular NACHT domain, and homologous PYD domain, which interact with PYD of adapter ASC. The CARD domain of ASC allows interaction with pro-caspase-1, which matures into caspase-1 upon NLRP3 inflammasome activation and oligomerization. (**b**): NLRP3 inflammasome activation in adipocyte invading ATM’s requires two signals. The first signal involves the priming signal, which is induced by endogenous cytokines or microbial components, such as lipopolysaccharide (LPS), leading to NF-κB-mediated upregulation of NLRP3 and pro-IL-1β. The second signal triggered by specific stimuli, PAMPs, and DAMPs, results in stresses, such as K^+^ efflux, mitochondrial dysfunction, and lysosomal disruption, which stimulates NLRP3 inflammasome formation. The activation of the NLRP3 inflammasome leads to procaspase-1 self-cleavage, generating active caspase-1, which in turn mediates IL-1β and IL-18 secretion and pyroptosis.

In ATM, NLRP3 inflammasome activation is regulated at both the transcriptional and post-translational levels. Over the past 10 years, a two-step activation model of priming and activation of NLRP3 inflammasome has been established [24]. The first signal in inflammasome activation involves the priming signal, which is induced by endogenous cytokines or microbial components, such as lipopolysaccharide (LPS), leading to NF-κB-mediated upregulation of NLRP3 protein, pro-IL-1β and pro-IL-18 [25]. Caspase-8 and FAS-mediated death domain (FADD) protein, and NOD1/2, are involved in the priming step [26]. The second signal is the inflammasome formation triggered by specific stimuli: PAMPs and DAMPs. When cells are stimulated, NLRP3 assembles by the NACHT domain to provide a scaffold for ASC oligomerization by the interaction between PYDs, and the oligomerized ASC interacts with caspase-1 via CARD homophilic interaction. The activation of NLRP3 inflammasome leads to procaspase-1 self-cleavage, generating the active caspase-1, which in-turn mediates the secretion of pro-inflammatory signals, IL-1β and IL-18. Active caspase-1 also induces Gasdermin-D-mediated plasma-membrane pore formation, osmotic swelling and pyroptosis, leading to a cascade of inflammation [27]. This classical NLRP3 inflammasome activation occurs in invading ATM’s resulting from an obesity-mediated alteration in adipose tissue microenvironment. Figure 1 summarizes the NLRP3 inflammasome activation in adipose tissue and its role in the maturation of pro-inflammatory signals IL-1β and IL-18.

### 2.1. Signal 1: Priming the NLRP3 Inflammasome

Before NLRP3 assembly and inflammasome activation, a priming stimulus by TLRs, NLRs, and cytokine receptors is required for NF-κB-mediated expression of NLRP3 protein, pro-IL-1β and pro-IL-18. Upon TLR binding by its agonists, several pathways involved in NLRP3 priming are successively activated over time, including the downstream transcription and activation of these receptors, which depend on several other molecules. The first pathway, referred to as transcriptional priming or late priming, relies on de novo protein synthesis, as several hours (>3 h) are required before actual NLRP3 assembly and activation happens [25]. The TIR domain-containing adapter-inducing interferon-β (TRIF), myeloid differentiation primary response 88 (MyD88), Fas-associated protein with death domain (FADD), caspase-8, and reactive oxygen species (ROS) participates in the NLRP3 priming. FADD is involved in the NF-κB signaling pathway and inhibits NF-κB activation by promoting apoptosis. Caspase-8 has a role in NF-κB activation through interaction with the inhibitor of nuclear factor kappa B (IKK) complex, which stimulates the NF-κB transcription and translocation [28]. In the resting state, the expression of NLRP3 and pro-IL-1β is low; both are highly transcriptionally upregulated downstream of TLR activation.

A second pathway was discovered recently, referred to as an early and intermediate priming pathway where NLRP3 basal expression levels are sufficient for inflammasome activation. This transcriptional-independent priming mechanism is effective 10 min to 1 h after TLR binding relies on mitochondrial ROS (mtROS) and can even bypass the requirement for TLR agonists [29,30]. This priming signal is mediated by signaling molecules downstream of TLRs and MyD88/IL-1 receptor-associated kinase 1 (IRAK-1). Phosphorylation of IPKA-1 leads to inflammasome activation, which is independent of the IKK complex [29].

### 2.2. Signal 2: Activating the NLRP3 Inflammasome

Various stimuli can activate NLRP3, including extracellular ATP, pore-forming toxins, heme particulate matter, and pathogen-associated RNA. NLRP3 cannot physically interact with its activators due to chemical and structural diversity. NLRP3 inflammasome activation signals can be diverse and depend on the cellular stresses that they cause.

#### 2.2.1. Conditions Causing Permeability to Ions

Activators, including pore-forming bacterial toxins like nigericin, ATP, particulate molecules, and crystals such as asbestos, silica, and uric acid, are known to induce cell permeability to ions, resulting in K^+^ efflux and Ca^2+^ signaling, which have been identified as critical events in NLRP3 inflammasome activation [31].

##### K^+^ Efflux

The role of K^+^ efflux in IL-1β maturation was first reported in 1994 [32]. A low intracellular K^+^ concentration triggers NLRP3 inflammasome activation [33]. Upon exposure to extracellular ATP, particulates, crystals, nigericin, etc., a decrease in cytosolic K^+^ is observed, and experimental modulation of extracellular K^+^ concentration correlates with inflammasome activation, suggesting its key role [34]. The mechanistic link, however, remains poorly understood with regard to conformational changes in NLRP3 oligomers induced by low cytosolic K^+^ concentration [35].

##### Ca^2+^ Signaling

The requirement of Ca^2+^ signaling in NLRP3 activation has been suggested by studies that show that the inhibition of Ca^2+^ signaling blocks NLRP3 inflammasome activation [36]. The main organelle for Ca^2+^ storage is ER, and it plays an important role in the maintenance of Ca^2+^ concentration [37]. Pharmacological inhibition of Ca^2+^ release channels on ER called inositol 1, 4, 5-triphosphate receptor (IP3R) or phospholipase C (PLC) attenuates Ca^2+^ mobilization and NLRP3 activation. The activation of IP3R is triggered by IP3, which in turn is a product of PLC-mediated phosphatidylinositol 4,5-bisphosphate (PIP2) cleavage, and this Ca^2+^ flux-associated NLRP3 activation also depends on various inflammatory stimuli. Apart from ER-mediated Ca^2+^ influx, lysosomal disruption following particulate phagocytosis may also contribute to a rise in cytosolic Ca^2+^ levels [38]. The mechanistic link between Ca^2+^ mobilization and NLRP3 activation is still unclear; however, production of mtROS due to mitochondrial damage by Ca^2+^ overload has been postulated as a trigger [39].

##### Na+ Influx and Cl^–^ Efflux

Some studies have postulated the role of Na^+^ influx [40] and intracellular Ca-efflux [41] in NLRP3 inflammasome activation. Na^+^ overload promotes water influx and cellular swelling leading to decreased K^+^ concentration and NLRP3 activation. Furthermore, inhibition of Cl^−^ efflux through volume-regulated anion channel (VRAC) prevents inflammasome activation, whereas the enrichment of anion channels called chloride intracellular channel (CLIC) in plasma membrane promotes Cl^−^ efflux and subsequent inflammasome activation [42].

#### 2.2.2. Reactive Oxygen Species (ROS) and Mitochondrial Dysfunction

One of the first identified stimuli of NLRP3 activation is reactive oxygen species (ROS), especially from mitochondria (mtROS) [43,44]. Agonists that cause mitochondrial dysfunction and ROS production increase NLRP3 inflammasome activation, and conversely, inhibitors preventing ROS production mitigate inflammasome activation [45]. Other studies have shown that NADPH oxidase 4 (NOX4) regulating carnitine palmitoyltransferase 1A (CPT1A) participates in increased fatty acid oxidation, which leads to NLRP3 inflammasome activation [46]. The precise role of ROS and the mechanism through which it activates the oligomerization of NLRP3 is not completely understood. However, some studies suggest a newly identified component of NLRP3 inflammasome called NIMA-related kinase 7 (NEK7), which can directly bind to LRR domain of NLRP3 and acts as a sensor of ROS needed for NLRP3 inflammasome assembly [47]. Other studies suggest a role of ROS in NLRP3 protein expression at the priming stage [25]. Mitochondrial has also been associated with the activation of NLRP3 inflammasome [45]. Studies also show that cardiolipin, a mitochondrion-specific phospholipid, binds to the LRR domain of NLRP3 and stimulates its assembly and activation [48]. Furthermore, some other mitochondrial molecules, including mitochondrial antiviral-signaling protein (MAVS) and mitofusin 2, have been implicated with NLRP3; however, the exact role remains to be determined [49,50].

#### 2.2.3. Lysosomal Damage

Several particulate alum, silica, asbestos, amyloid-β, cholesterol crystals, calcium crystals, etc. stimulate the NLRP3 inflammasome activation in macrophages through damage in lysosomes after phagocytosis, resulting in the release of lysosomal contents into the cytosol. Lysosomal contents, such as cathepsin B, have a role in inflammasome activation at priming (pro-IL-1β synthesis) and inflammasome assembly stage [51]. The mechanism of particulate-induced lysosomal rupture and inflammasome activation is still unclear; but some studies suggest that cytosolic release of cathepsins triggers K^+^ efflux which is required for NLRP3 inflammasome activation [34].

### 2.3. Post-Translational Modifications of NLRP3 and Associated Proteins as Regulators of NLRP3-Inflammasome Activation

Inflammasomes directly recognize the signal for activation through their sensors and/or indirectly by sensing the cellular environment, as discussed earlier. In addition, several post-translational modifications (PTM), such as phosphorylation, ubiquitination, alkylation, and s-nitrosylation, in NLRP3 and associated proteins play a critical role in the NLRP3 inflammasome activation. The regulation of NLRP3 inflammasome is important to prevent the detrimental effects of the uncontrolled activation, as in inflammatory diseases like metabolic syndrome. Table 1 summarizes different PTMs associated with NLRP3 inflammasome activation.

### 2.4. NLRP3-Interacting Proteins and Their Effect on Inflammasome Activation

Multiple NLRP3-interacting proteins have been proposed to regulate the activity of NLRP3 inflammasome. One such critical regulator is NEK7, which through several pathways, such as ROS signaling, K^+^ efflux, and lysosomal destabilization, plays an important role in NLRP3 inflammasome activation [71,72]. NEK7 is a multifunctional kinase whose role in recent years has been demonstrated in mitotic spindle assembly, mitochondrial regulation, intracellular protein transport, and DNA repair [73,74], which indicates its involvement in the development of cancer [75]. Recent studies demonstrate that the N-terminal region of NEK7 interacts with the C-terminal LLR and NOD region of NLRP3 and helps in maintaining the integrity of the cellular microtubule network, which is important in NLRP3 inflammasome activation [76]. A similar protein called microtubule-affinity regulating kinase 4 (MARK4) binds directly to NLRP3, promotes its recruitment to mitochondria, and drives it to the microtubule-organizing center. The importance of MARK4 for inflammasome activation is established as disruption in NLRP3-MARK4 interaction or loss of MARK4, which alters NLRP3 inflammasome activation [77].

Heat shock protein 90 (HSP90), along with its co-chaperone protein called suppressor of G-Two allele of skp1 (SGT1), binds with the LRR domain of NLRP3 and protects it from degradation by both the proteasome and autophagy [78]. Several other HSP proteins, such as HSP60, HSP70, and HSP27, have a strong impact (both positive and negative) on inflammasome activation, which can be used to modulate the inflammation in metabolic disorders [79]. The interaction of an oxidative sensor called Thioredoxin-Interacting Protein (TXNIP), with NLRP3 in a ROS-induced mechanism, results in the subsequent activation of NLRP3 inflammasome [80]. Similarly, Guanylate binding protein 5 (GBP5) appears to promote NLRP3-mediated ASC oligomerization, specifically in live bacteria and soluble priming agents, by binding to the pyrin domain of NLRP3 [81]. Another important protein, which is activated by double-stranded RNA (dsRNA) upon viral infections, called RNA-activated protein kinase (PKR), is involved in regulating the NF-kB pathway. However, its role in NLRP3 inflammasome activation has been contradictory [82,83]. Additional studies to understand the role of these NLRP3-interacting proteins in inflammasome assembly and activation are needed. 

## 3. NLRP3 Inflammasome in Obesity-Associated Metabolic Syndrome

Metabolic syndrome is a multifactorial pathophysiological disorder characterized by inflammation in tissues such as adipose, liver, and pancreatic islets. Infiltration of macrophages, dendritic cells, T cells, B cells, and NK cells in these tissues accompanied by various cytokines and chemokines leads to low-grade tissue inflammation. Vandanmagsar et al. reported a crucial role of NLRP3 in this obesity-induced inflammatory disorder where metabolic DAMPs, such as excess ATP, glucose, ceramides, reactive oxygen species, oxidized LDL, uric acid, as well as crystals of cholesterol and monosodium urate, leads to NLRP3 and IL-1β mediated pro-inflammatory response. This results in insulin resistance and metabolic syndrome [3]. Increased expression of NLRP3 and IL-1β have been observed in visceral and subcutaneous deposits in obese individuals, which has also been confirmed by genetic studies [84,85]. Besides, lower gene expressions of these two proteins were observed in response to calorie restriction, exercise, and weight loss through bariatric surgery [86,87], which indicates that obesity-induced metabolic syndrome and NLRP3 inflammasome activity are closely associated. However, some studies suggest that obesity-mediated inflammation and expression of adipose tissue inflammatory markers is independent of NLRP3 inflammasome activation [88].

### 3.1. Metabolic Regulators of NLRP3 Inflammasome Activation

**Figure 2:**
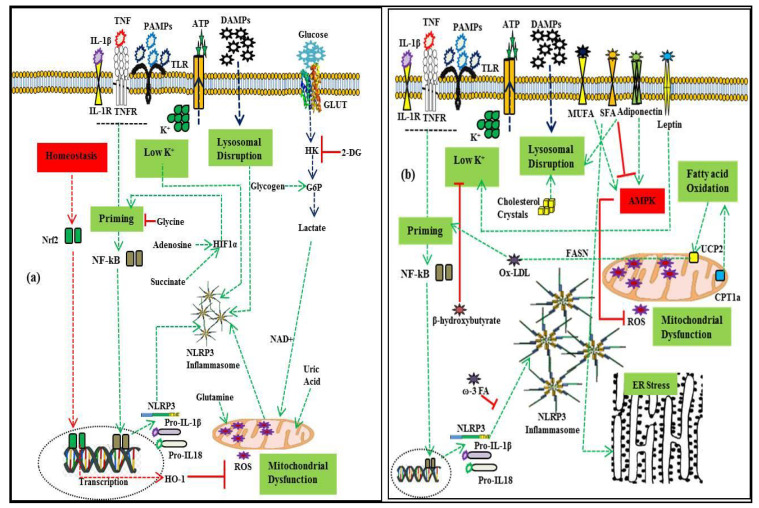
Metabolic regulation of NLRP3 inflammasome activation. (**a**): Regulation by glycolytic flux and amino acid metabolism. As a part of homeostatic regulation, the priming signal activates nuclear factor erythroid 2-related factor 2 (Nrf2), which promotes heme-oxygenase-1 (HO-1), mitigating the effect of mtROS. Increased production of succinate enhances the transcription of pro-IL1β through hypoxia-inducible factor (HIF-1α). Glycolytic flux promotes inflammasome activation which is inhibited by 2-deoxy-D-glucose (2-DG). Glycine enhances Nrf2-mediated mitigation of inflammasome activity. Glutamine and uric acid enhance it through mtROS. (**b**): Regulation by adipokines and lipid metabolism. The activity of fatty acid synthase (FASN) promotes priming, while omega-3 fatty acid inhibits inflammasome assembly. Saturated fatty acids promote lysosomal disruption and ER stress, while monounsaturated fatty acids promote AMP-activated protein kinase, which in turn inhibits mtROS production. Cholesterol crystals promote inflammasome activation through lysosomal disruption. Similarly, carnitine palmitoyl-transferase 1A (CPT1A) promotes inflammasome activation, and β-hydroxybutyrate inhibits it by suppressing K^+^ efflux. Adiponectin acts as an initiator of AMPK-autophagy inhibition of mtROS and K^+^ efflux, while leptin enhances NLRP3 inflammasome activation. Green lines depict the promotion of inflammasome activation, while red lines depict inhibition.

Cellular metabolites, carbohydrates, and lipids in their many forms can act as regulators of the NLRP3 inflammasome. Research relating to the modulation of the NLRP3 inflammasome by diet and fatty acid-induced obesity will open new avenues for nutrient-sensitive metabolic inflammation. Figure 2 provides an overview of the metabolic regulation of NLRP3 inflammasome activation.

#### 3.1.1. Lipids

Intake of saturated fatty acids (SFA’s) such as palmitic acid, is strongly associated with obesity and has been associated with insulin resistance and inflammatory disorder in humans [89]. Palmitic acid, like other SFA’s, inactivates AMPK, which impairs autophagy, and induces mtROS production, and in turn leads to activation of the NLRP3 inflammasome and IL-1β mediated insulin resistance [46]. Additionally, crystalline palmitic acid induces lysosomal membrane rupture in macrophages and elicits ER stress, which contributes to inflammasome activation [90]. In contrast, oleic acid, an unsaturated fatty acid, inhibits the effects of palmitic acid and helps in reducing NLRP3 inflammasome activation by reducing ER stress and promoting AMPK activation [91]. Similarly, long-chain polyunsaturated fatty acids (PUFAs), such as omega-3 FAs, inhibits caspase-1 activation through G-protein receptor 120 (GPR120)-mediated β-arrestin 2 bindings of NLRP3 blocking NLRP3 inflammasome assembly and activation [92]. Moreover, adipocytes under stress release lysophosphatidylcholine, which interacts with GPR132 and triggers diverse intracellular events needed for the full NLRP3 inflammasome activation [93]. Cholesterol, another type of lipid, has also been implicated in the disruption of the lysosomal membrane and NLRP3 inflammasome activation [94]. Oxidized low-density lipoprotein (oxLDL), a cholesterol carrier that promotes atherosclerosis, was shown to activate NF-κB and thus promote upregulation of NLRP3 [95]. Its activity is suppressed by fibronectin domain-containing protein 5 (FNDC5) by blocking NF-κB activation in an AMPK-dependent manner [96].

#### 3.1.2. Carbohydrates

Glycolysis, an important metabolic process, plays a central role in macrophage activation, as proinflammatory macrophages rapidly increase their rate of glycolysis; hence, it is considered to be a major regulator of the NLRP3 inflammasome. Hyperglycaemia can stimulate NLRP3 inflammasome activation in human adipose tissue by upregulating the expression of TXNIP [97]. Glycolysis inhibitors 2-deoxy-D-glucose (2-DG) and aminooxy acetic acid also attenuate NLRP3 inflammasome activation [98]. Other enzymes of the glycolytic pathway such as hexokinase, pyruvate kinase M2 (PKM2), and tyrosine-protein kinase (Syk), have also been implicated as regulators of inflammasome activation [99,100,101].

#### 3.1.3. Amino Acid and Nucleotide Metabolism

An increase in glutamate during ischemic conditions of glucose deprivation and hypoxia induces ER stress, Ca^2+^ influx, and TXNIP expression, resulting in NLRP3 inflammasome activation [102]. The amino acid glycine was found to attenuate IL-1β production by decreasing the expression of NLRP3 inflammasome components [103]. The best-characterized stimuli of the NLRP3 inflammasome is ATP (nucleotide-containing DAMP), which regulates it in a manner beyond the initial upregulation of IL-1β by NF-κB [104]. Another nucleotide pathway metabolite, namely enzyme xanthine oxidoreductase (XOR), has been shown to partially attenuate IL-1β secretion by decreasing mtROS [105]. Additionally, crystallized uric acid, in a MyD88- and/or mtROS-dependent manner, promotes the activation of the NLRP3 inflammasome [106].

#### 3.1.4. Lipopolysaccharides (LPS)

One of the most potent PAMPs for priming the NLRP3 inflammasome is the endotoxin located on the outer membrane of Gram-negative bacteria called LPS. LPS increase expression of pro-IL-1β and NLRP3 through TLR4- and NF-kB-dependent pathways at the transcriptional and post-translational levels and stimulates NLRP3 deubiquitination required for NLRP3 inflammasome activation. In obese and diabetic states, the circulating level of LPS increases due to changes in gut microbiota composition and increased gut permeability, which in turn is absorbed by the macrophages and adipocytes, eliciting an inflammatory response [107].

#### 3.1.5. Adipokines

Adipokines, the cell-signaling proteins secreted by the adipose tissue, plays an important role as regulators of NLRP3 inflammasome activation. Adiponectin, an adipose tissue-derived hormone, attenuates NLRP3 inflammasome by AMPK, autophagy, and/or NF-kB pathways [108]. Meanwhile, via feedback loop, adiponectin expression and secretion are downregulated by IL-1β [109]. Leptin, another adipose tissue hormone highly expressed in WAT, has also been implicated in NLRP3 inflammasome activation, as it promotes IL-18 secretion through the activation of caspase-1 [110]. Resistin, an adipose-secreted hormone known to impair glucose tolerance, stimulates synthesis, and secretion of inflammatory cytokines, and induces NLRP3 inflammasome activation [97]. TNF-α, a multifunctional adipokine that is increased in obesity, is a potent endogenous NLRP3 inflammasome priming signal driving age-related inflammation [111]. TNF-α binds to its receptor and activated signaling pathways, such as NF-κB and JNK, and has been implicated in MetS by stimulating inflammation through the generation of ROS and inducing transcriptional-mediated pathways. Table 2 provides an overview of the regulation of NLRP3 inflammasome activation by key adipokines.

## 4. NLRP3 Inflammasome and Disorder in Metabolic Homeostasis

**Figure 3:**
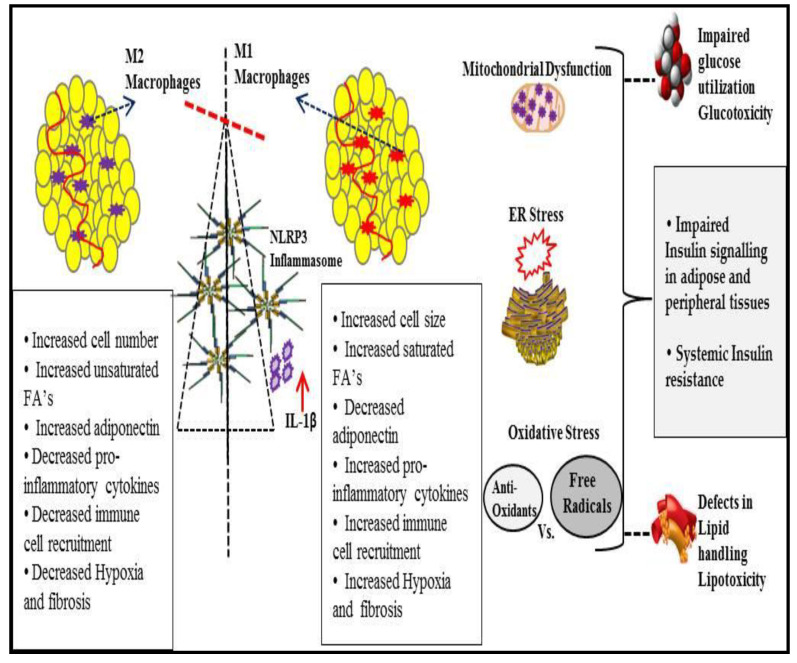
Overview of obesity-associated metabolic inflammation and insulin resistance. NLRP3 inflammasome activation as a consequence of obesity-mediated immunological and metabolic dysregulation, including changes in adipose tissue-resident immune cells, impaired adipogenesis, decreased anti-inflammatory adipokines, hypoxia, fibrosis, etc., and helped by oxidative and ER stress, leads to a glucotoxicity, lipotoxicity, and systemic insulin resistance.

Excess nutrients, lipolysis, gluconeogenesis, etc., in obesity induce mitochondrial dysfunction, oxidative stress, and ER stress, which in turn stimulates stress-responsive molecules, such as JNK and IKKβ, leading to exacerbation of hyperlipidemia and hyperglycemia. Inflammation in adipose tissues, mediated by these stress-induced signals, induces insulin resistance. Furthermore, adipocytes and infiltrated immune cells mediate inflammation via proinflammatory adipokines. This section summarizes the consequences of NLRP3 inflammasome activation in obesity-induced inflammation and insulin resistance (Figure 3).

In obesity, adipose tissue responds rapidly to changes in nutrient excess via an increase in adipocyte size (hypertrophy) and number (hyperplasia) to play its major role in energy homeostasis. Adipose tissue remodeling accelerates in the obese state and is mediated by the process of adipogenesis, in which progenitor cells differentiate into mature adipocytes. Defects in this process contribute to the impairment in recruitment of fresh adipocytes and in turn lead to adipocyte enlargement. NLRP3 inflammasome is associated with the downregulation of adipogenesis, leading to a state of adipocyte hypertrophy [129]. This expansion may lead to, for example, adipocyte hypoxia and death, and/or enhanced chemokine secretion [130]. This results in macrophages aggregating and forming a crown-like structure around necrotic adipocytes, which plays a pivotal role in adipose tissue remodeling [131], in which a transformation occurs in the polarization states of macrophages from an anti-inflammatory M2 state to a proinflammatory M1 state [132]. This also results in an increase in circulating levels of monocytes that infiltrate WAT and aggravate the inflammation. It has been shown that expression of monocyte chemoattractant protein-1 (MCP-1), a key regulator of ATM influx to WAT, reduces as a result of HFD-fed mice with caspase-1 deletion [97].

Chronic adipocyte hypoxia stimulates transcription factors called hypoxia-inducible factor 1 (HIF-1) and NF-kB and has been linked to adipose tissue dysfunction, namely ER stress [133], oxidative stress and production of ROS [134], and regulation of adiponectin and leptin expression respectively [135,136], which ultimately results in insulin resistance [137]. Insulin resistance is considered as the primary causative factor in the development and manifestation of MetS. Normally, the activation of insulin by binding to its receptor in many tissues of the body, including the liver, muscle, adipose tissue, and vascular endothelium, will initiate a cascade of event. This process begins with the binding of insulin to its receptor and stimulates autophosphorylation on tyrosine residues of a set of insulin receptor substrate proteins. These proteins activate a lipid kinase, namely phosphatidylinositol 3-kinase (PI3K). PI3K converts the phosphoinositide PIP2 to PIP3, a lipid species that is specifically recognized by proteins with pleckstrin-homology (PH) domains, notably the protein kinase AKT/PKB which phosphorylates a number of targets, including Forkhead box protein O1 (FOXO1), a factor that regulates the expression of insulin-sensitive genes [138]. Insulin signaling affects the energy levels of the body via several mechanisms, including activating glycogen synthase and decreasing the transcription of enzymes phosphoenolpyruvate carboxykinase (PEPCK) and the glucose-6 phosphatase (G6Pase) and via suppression of FOXO1 and activating sterol regulatory element-binding protein 1 (SREBP1), leading to the suppression of glucose production and hepatic gluconeogenesis [139]. NLRP3 activation impairs insulin sensitivity in dietary-induced obesity. Disruption of phosphatidylinositol 3-kinase-protein kinase B (PI3K-Akt) signaling plays a major role in NLRP3-mediated insulin resistance [140]. Moreover, IL-1β alters phosphorylation of the insulin receptor substrate (IRS) [141]. Taken together, insulin resistance affects many tissues, causing dysregulation of metabolism and leading to hyperglycemia, hyperinsulinemia, and hypertriglyceridemia, all of which contribute to a vicious cycle of MetS manifestations.

Oxidative stress is the state of imbalance between oxidative and antioxidative systems at the biological level, generating excessive oxidative free radicals and ROS. High levels of ROS lead to cellular dysfunction by altering the metabolism of proteins, lipids, and nucleic acid, which results in activation of the immune system and inflammation [142]. Oxidative stress stimulates the expression of enzymes responsible for catalyzing the hydro-peroxidation of polyunsaturated fatty acids (12/15-lipoxygenase (12/15-LOX)). A high level of 12/15-LOX causes endoplasmic reticulum stress and unfolded protein response (UPR) [143]. In addition, releasing proinflammatory mediators as a result of stress reduces the expression of eNOS and NO production and suppresses cGMP and PGC-1α (peroxisome proliferator-activated receptor coactivator 1 alpha), in which reduces mitochondria biogenesis [144].

Oxidative stress was found to be significantly associated with MetS and its components via several mechanisms, either as a cause or a consequence [145]. One of these mechanisms lies in the electron transport chain (ETC). An increase in the metabolic load of the mitochondria leads to overactive ETC, which can result in overproduction of ROS as by-products, contributing to damage to the proteins, DNA, and lipids in mitochondria [146]. NLRP3 inflammasome activation also has deleterious effects on lipid synthesis and utilization by adipocytes as fat oxidation rate, mitochondrial energy dissipation, and lipolysis diminish. NLRP3 activation down-regulates expression of growth differentiation factor-3 (GDF3) and monoamine oxidase A (MAOA), implicated in catecholamine catabolism, leading to a reduction of glycerol and free fatty acids (FFAs). NLRP3 ablation restores the proper expression of lipolytic enzymes and reverses age-related catecholamine degradation [147].

## 5. Inhibition of NLRP3 Inflammasome Activation

The role of NLRP3 inflammasome activation in the exacerbation of obesity-mediated metabolic disorders and many other diseases opens new avenues for treating or relieving complications associated with these disorders. The first to be targeted were the final products of the activation, namely IL-1β and IL-18, with, for example, IL-1β antibodies and recombinant IL-1β receptor antagonists, such as canakinumab and anakinra, respectively. IL-1β and IL-18 are both potent immune modulators that cascade immune response, and have been implicated in many inflammatory processes, and at higher levels could have disastrous consequences if left uncontrolled. IL-1β signaling results in the production of proinflammatory cytokines, such as TNF-α, CRP, IL-8, and IL-6, and chemokines that attract macrophage invasion, such as MCP1 (monocyte chemoattractant protein 1) and MIP2 (macrophage inflammatory protein 2). IL-1β and IL-18-targeted therapies have been used to treat many autoinflammatory diseases; however, this may lead to unintended immunosuppressive effects and the risk of opportunistic infections. Instead, therapies targeting upstream of IL-1β production and specific NLRP3 inflammasome inhibitors might prove efficient and improve safety. Table 3 provides an overview of the potential inhibitors of NLRP3 inflammasome activation.

## 6. Conclusions

Several studies have shown that obesity is associated with low-grade systemic inflammation that contributes to insulin resistance and metabolic disorders, associated by changes in adipose tissue-resident immune cells. This inflammation in adipose tissue is likely initiated by a classical two-step activation of the NLRP3 inflammasome with metabolic insults stimulating these two signals. Metabolic insults, such as increased SFAs, proinflammatory adipokines, hyperglycemia, etc., promote K^+^ efflux, mitochondrial dysfunction and ROS production, lysosomal disruption, etc., leading to NLRP3 inflammasome activation and caspase-1-mediated IL-1β and IL-18 secretion and pyroptosis, which in turn mediate a systemic cascading inflammatory response. Meanwhile, several PTMs in NLRP3 and related proteins, such as phosphorylation, ubiquitination, alkylation, and s-nitrosylation, also play a critical role. In this process, many cellular metabolites as well as adipokines act as regulators.

Adipose tissue remodeling as a response to changes in nutrient excess in obesity, followed by adipocyte hypoxia and enhanced chemokine secretion, leads to NLRP3-mediated adipose tissue inflammation, which ultimately results in the impairment of the insulin signaling pathway and insulin resistance, the primary causative factors in the development and manifestation of metabolic syndrome. Research on the modulation of the NLRP3 inflammasome via diet and fatty acid-induced obesity will open new avenues for treating or relieving complications in metabolic inflammatory disorders. At the same time, the beneficial aspects of NLRP3 inflammasome inhibition on adipose tissue inflammation and metabolic health need to be further investigated in light of these insights and the possible side effects of immunosuppression.

## Figures and Tables

**Table 1:** Regulation of NLRP3-inflammasome activation through post-translational modification of NLRP3 and associated proteins.

Sl#	PTM	Protein	Modification Site	Regulatory Enzyme	Role in Regulation of NLRP3 Inflammasome	Ref.
1	Phosphorylation	NLRP3	Y861 (LRR domain)	Protein tyrosine phosphatase non-receptor 22 (PTPN22)	Dephosphorylation at Typ861 residue leads to efficient NLRP3 inflammasome activation and Il-1β secretion. ^●^	[52]
2			Ser291 or Ser295 (NACHT domain)	Protein Kinase A (PKA)	Cholesterol catabolism leads to an increase in intracellular cAMP levels, activating PKA, which phosphorylates NACHT domain at Ser291 or Ser295 site, leading to NLRP3 degradation and inhibiting inflammasome activation. ^●^	[53,54]
3			Ser5 (PYD domain)	Protein Phosphatase 2A (PP2A)	Dephosphorylation of Ser5 site at the PYD domain of NLRP3 protein by PP2A promotes NLRP3-ASC interaction, which is required for inflammasome assembly. ^●^	[55]
4			Ser194 (NACHT domain)	Jun N-Terminal Kinase (JNK)	TLR ligands phosphorylate the Ser194 residue in the NACHT domain of NLRP3 protein in a JNK1-dependent manner to facilitate inflammasome assembly. ^●^	[56]
5			Ser295 (NACHT domain)	Protein Kinase D (PKD)	Activated PKD phosphorylates the Ser293 residue in the NACHT domain, promoting its release from mitochondria-associated membranes (MAMs) to the cytoplasm, thereby facilitating inflammasome assembly and maturation. ^●^	[57]
6	Ubiquitination	NLRP3	LRR domain at an unknown site	BRCA1/BRCA2-containing complex 3 (BRCC3)	LPS stimulation induces NLRP3 deubiquitination at an unknown site of the LRR domain in a BRCC3-mediated manner to promote activation. ^●^	[58]
7			Lys689 (LRR domain)	F-Box L2 (FBXL2)	FBXL2, a ubiquitin E3 ligase, interacts with NLRP3 protein at lysine689 residue of the LRR domain, leading to its degradation. ^●^	[59]
8			PYD domain at an unknown site	Tripartite motif-containing protein31 (TRIM31)	TRIM31, a ubiquitin E3 ligase, promotes its K48-linked ubiquitination at the PYD domain leading to proteosomal degradation, and is a part of the feedback suppressor of the inflammasome. ^●^	[60]
9			NACHT and LRR domains at unknown sites	Membrane-associated RING-CH-type finger protein 7 (MARCH 7)	MARCH 7, another ubiquitin E3 ligase, promotes ubiquitination and degradation of NLRP3 at both NACHT and LRR domains in response to stimulation of dopamine D1 receptor (DRD1), leading to NLRP3 inflammasome inhibition. ^●^	[61]
10			NACHT domain at an unknown site	Ariadne Homolog 2 (ARIH2)	ARIH2, another ubiquitin E3 ligase, induces K48 ubiquitination at the NACHT domain of NLRP3 and acts as an endogenous negative regulator of NLRP3 inflammasome activation. ^●^	[62]
11			Unknown domain	Pellino2 (PEL2)	Pellino2, an E3 ubiquitin ligase, facilitates the activation of NLRP3-inflammasome by promoting the ubiquitination of NLRP3 at an unknown domain during the priming stage. ^●^	[63]
12		ASC	CARD domain	Tumor necrosis factor receptor-associated factor 3 (TRAF3)	Ubiquitination of CARD domain of ASC at K174 residue with K63 chains stabilizes it and promotes NLRP3-inflammasome assembly. ^●^	[64]
13			Unknown domain	Ubiquitin Specific Peptidase 50 (USP50)	USP50 deubiquitinates Lys-63 at an unknown domain of ASC and promotes speck formation and oligomerization, helping in NLRP3-inflammasome activation. ^●^	[65]
14			Unknown domain	Linear Ubiquitin Assembly Complex (LUBAC)	LUBAC has been implicated as a key driver of the nuclear translocation of NF-kB and hence plays an important role in NLRP3-inflammasome activation. ^●^	[66]
15		Caspase-1	CARD domain	Cellular inhibitor of Apoptosis proteins 2 (clAP-2)	clAP-2 mediates the polyubiquitination of the CARD domain of caspase-1 leading to its activation. ^●^	[67]
16		Pro-IL-1β	Unknown domain	A20	A20, a ubiquitin modifying enzyme, is an NFkB inhibitor that reduces pro-IL-1β K63 ubiquitination and maturation and hence inhibits NLRP3-inflammasome activation. ^●^	[68]
17	Alkylation		Unknown domain	3,4-methylenedioxy-β-nitrostyrene (MNS)	NLRP3-alkylating agents like MNS reduce the ATP binding affinity of NLRP3, which is required for NLRP3-ASC association and hence negatively regulates NLRP3 inflammasome activation. ^●^	[69]
18	S-Nitrosylation		LRR domain	Inducible nitric oxide synthase (iNOS)	Expression of iNOS by prolonged exposure to LPS leads to the production of NO. This leads to the S-nitrosylation of the LRR region of NLRP3, preventing its oligomerization. ^●^	[70]

● and ● depict, respectively, the promotion and inhibition of inflammasome activation.

**Table 2:** Role of adipokines in the regulation of NLRP3 inflammasome activation.

Sl#	Adipokine	Regulation of Inflammasome Activation in Metabolic Disorder	Ref.
1.	Adipocyte fatty acid-binding protein 4 (FABP-4)	Control NLRP3 inflammasome activation through downregulating mitochondrial uncoupling protein-2 (UCP2) expression	[112]
2.	Adipsin	Critical as a complement system component in vascular complications of metabolic disorder	[113]
3.	Angiotensinogen and Angiotensin II	Linked to ER stress-induced NLRP3 inflammasome activation	[114]
4.	Apelin	Inhibits NF-kB pathway and inflammasome activation helping in vasodilation	[115]
5.	C-reactive protein (CRP)	Up-regulates NF-κB activity, thereby promoting IL-1β mediated atherosclerosis	[116]
6.	Fibroblast growth factor 2 (FGF-2)	Enhances endothelial adhesion molecule (EAM) expression involved in NLRP3-mediated endothelial dysfunction	[117]
7.	Hepatocyte growth factor (HGF)	Promotes inhibition through up-regulation of adiponectin in adipocytes	[118]
8.	Intercellular adhesion molecule 1 (ICAM-1)	Involved in NF-κB mediated TNF-α signaling pathway and endothelial inflammation	[119]
9.	Lipoprotein lipase (LPL)	Plays a central role in triglyceride and phospholipid hydrolysis, the products of which could elicit pro- or anti-inflammatory responses in endothelial cells	[120]
10.	Matrix metalloproteinases (MMPs)	Involved in cartilage degeneration and NLRP3-mediated synovial inflammation in osteoarthritis	[121]
11.	Monocyte chemoattractant protein 1 (MCP-1)	Key chemokine that regulates the migration and infiltration of adipose tissue via by monocyte/macrophages	[122]
12.	Omentin 1	Involved in inhibition of the TXNIP/NLRP3 signaling pathways in adipose tissue	[123]
13.	Perilipin 1	Involved in lipid metabolism homeostasis and inhibits the NF-κB inflammatory pathway	[124]
14.	Plasminogen activator inhibitor 1 (PAI-1)	Plays an important role in regulating ROS-mediated fibrinolysis	[125]
15.	Serum amyloid A	Promotes NLRP3 inflammasome activation via the cathepsin-sensitive pathway	[126]
16.	Vaspin	A visceral adipose tissue-derived serpin that can regulate the PI3K/AKT signaling pathway and improve myocardial function by inhibiting NLRP3 expression	[127]
17.	Visfatin	Visfatin, a pre-B-cell colony-enhancing factor, is involved in TL4-mediated endothelial dysfunction and vascular inflammation	[128]

**Table 3:** Potential pharmacological inhibitors of NLRP3 inflammasome activation.

Sl#	Compound	NLRP3 Inflammasome Inhibition	Inflammasome Target	Target Disease	Ref.
1.	MCC950	Sulfonylurea compound that block nigericin-induced NLRP3 inflammasome activation by inhibiting chloride efflux	NACHT domain of NLRP3	Atherosclerosis, myocardial infarction, colitis, and skin and airway inflammation	[148]
2.	Tranilast	Tryptophan derivative that inhibits NLRP3-NLRP3 interaction and subsequent ASC oligomerization		Gouty arthritis and cryopyrin-associated periodic syndrome (CAPS)	[149]
3.	OLT1177	β-sulfonyl nitrile compound that inhibits NLRP3-NLRP3 interaction		Gouty arthritis and cryopyrin-associated periodic syndrome (CAPS)	[150]
4.	Oridonin	Prevents NLRP3-NEK7 interaction by binding to Cys 279 residues at the NACHT domain		Alzheimer’s disease and cancer	[151]
5.	CY-09	Analog of a cystic fibrosis transmembrane conductance regulator (CFTR) channel inhibitor that impairs NLRP3 ATPase.		Gout, atherosclerosis, and neurodegenerative diseases	[148]
6.	MNS	Impairs ATPase activity of NLRP3 by covalently modifying Cys residues at the NACHT domain		Gout, atherosclerosis, and neurodegenerative diseases	[69]
7.	BOT-4-one	NLRP3 alkylation by BOT-4-one leading to impaired ATPase activity in NACHT domain		Inflammatory skin diseases	[152]
8.	IFN39	Impairs ATPase activity of NLRP3 by binding at the NACHT domain		Inflammatory bowel disease	[153]
9.	Bay 11-7082 and Parthenolide	Inhibit ATPase activity of NLRP3, which is required for activation of Caspase-1	Caspase-1	Systemic lupus erythematosus	[154]
10.	Pralnacasan and Belnacasan	Selectively inhibit Caspase-1 protease activity		Rheumatoid arthritis	[155]
11.	GKT137831 and VAS-2870	NOX4-mediated inhibition of caspase-1 activation		Systemic sclerosis and pulmonary fibrosis	[156]
12.	Etomoxir	CPT1A-mediated inhibition of caspase-1 activation		Congestive heart failure and psoriasis	[157]
13.	Z-VAD-FMK	Binds to the catalytic site of caspase proteases and inhibits their activity		Granulosa cell apoptosis	[158]
14.	Necrosulfonamide	Alkylating compound binds to Gasdermin D, thereby preventing pyroptotic pore formation and cell lysis	Gasderimin D	Hemorrhagic necrosis	[159]
15.	Glyburide	Sulfonylurea-containing compound inhibits ATP-sensitive potassium channels in pancreatic β-cells	NLRP3 (indirectly)	Type 2 diabetes	[160]
16.	16673-34-0	Sulfonyl compound in glyburide synthesis pathway that inhibits inflammasome activity in the heart		Heart diseases	[161]

## Data Availability

Not applicable.
